# The relationship between disgust sensitivity and serum cytokine levels in non-pregnant women: a generalization of previous results on pregnant population

**DOI:** 10.1016/j.bbih.2026.101374

**Published:** 2026-09-22

**Authors:** Daniela Dlouhá, Martin Hill, Josef Včelák, Šárka Kaňková

**Affiliations:** a Department of Philosophy and History of Science, Faculty of Science, Charles University, Viničná 7, Prague 2, 128 44, Czech Republic; b Department of Steroids and Proteofactors, Institute of Endocrinology, Národní 8, Prague, 110 00, Czech Republic; c Department of Molecular Endocrinology, Institute of Endocrinology, Národní 8, Prague 1, 110 00, Czech Republic

**Keywords:** Immune function, Disgust, Moral disgust, Pathogen avoidance, Behavioral immune system, Compensatory prophylaxis, Social immunity, Physiological immune system, Mental health

## Abstract

The behavioral immune system functions via the emotion of disgust, which triggers avoidant reactions that protect the individual from pathogens. The behavioral immune system is closely linked with the physiological immune system; their mutually compensatory and preparatory functions are discussed. A recent study on pregnant women supported the notion that disgust increases when the physiological immune system is not sufficiently activated. The aim of this current study was to confirm these results on 78 healthy non-pregnant women, measuring immune activity via multiplex immunoassay and disgust sensitivity via the Disgust Scale Revised (DS-R), Three Domains of Disgust Scale (TDDS) and Tactile Disgust Stimuli Set, as well as Perceived Vulnerability to Disease. We showed a significant negative association between DS-R contamination disgust and IL-1β, IL-2, IL-6, IL-8 and TNF-α and DS-R core disgust and MCP-1, suggesting the possibility of a compensatory relationship between the systems. Several cytokines were also negatively associated with TDDS moral disgust (e.g. IL-1β, IL-2, IL-5, TNF-α). Simultaneously, we observed positive associations between IL-2, IL-5, IL-10 and IL-12 and the DS-R core disgust. The role of these cytokines in chronic respiratory conditions and their associations with mental health provide suggestions for possible interconnections between the research areas of disgust, respiratory and mental health. We found no significant association with tactile disgust and perceived vulnerability to disease. Our findings could thus support both the adaptive function of disgust as well as its links to mental health and point to the complexity of the behavioral immune system.

## 1 Introduction

The body's physiological immune system (PIS) is a complex network of organs, cells and proteins that can facilitate (amongst others) an immune reaction, which protects the body against pathogens. However, this activation of the PIS has a number of drawbacks; it is quite energetically costly and the individual is weakened during the course of the reaction. Because it is activated after the individual comes into contact with the pathogen, it would seem beneficial if the contact could in some way be prevented and the system would not have to activate as often. That is the role of the behavioral immune system (BIS; Schaller and Duncan, 2007) and its affective component - the emotion of disgust.

The BIS is a series of mechanisms that involve a perceptive, cognitive, affective and behavioral component. In the perceptive component, cues from all sensory modalities (visual, auditory, olfactory, gustatory and tactile) enter the cognitive evaluation after which the affective reaction, disgust, is elicited and modulated. One of the signs of an activated BIS is that we feel disgusted upon noticing stimuli that can pose a pathogen threat. The disgust reaction then in turn facilitates a behavioral reaction – avoidance of the stimuli and of the pathogen threat (Schaller and Park, 2011). The BIS is functionally flexible, with the affective and behavioral response being modulated depending on a range of external and internal factors and has many health and social implications (Schaller and Park, 2011). The presence of pathogens in the environment is considered one of the external factors that may affect the BIS activity (Stevenson et al., 2021), along with age (Curtis et al., 2004), reproductive state (Dlouhá et al., 2024) or PIS activity (see below).

The activity of these two systems seems to be closely integrated, though the exact relationship is not yet clear. One such integration is the basis of a hypothesis stating that when the immune system is suppressed or less active, the activity of the behavioral immune system should be elevated to compensate. This premise is also known as the compensatory prophylaxis hypothesis (Fessler and Navarrete, 2003). Several studies have focused on testing this hypothesis. Some showed that disgust was elevated in people who reported being more frequently sick (Stevenson et al., 2009), recently sick (Dlouhá et al., 2023), more vulnerable to disease (Sarolidou et al., 2020), lived in an environment with higher risk of infection (Cepon-Robins et al., 2021; Hlay et al., 2021; Skolnick and Dzokoto, 2013) or faced a pandemic threat (Kaňková et al., 2023). A study also showed that recently ill people reacted more negatively to faces displaying disease cues (Miller and Maner, 2011), but it has not been successfully replicated (Tybur et al., 2020). Additionally, a number of other studies did not show support for aspects of this hypothesis, specifically that frequency of illness in childhood didn't impact disgust sensitivity (de Barra et al., 2014) and neither did the use of immunosuppressive medication, which even had an opposite effect (Oaten et al., 2016).

While these studies tested the relationship between the activity of the BIS and the PIS, the indicators of immune system activity were only based on self-reported information. A study, which did not directly focus on the immune system activity, nonetheless showed support for the compensatory relationship via finding a positive association between disgust sensitivity and levels of steroids with immunosuppressive effects during pregnancy (Kaňková et al., 2024). However, while this study had the advantage of using biomarkers, they were still only indirectly related to immune system activity.

Finally, a smaller body of studies tested this relationship using immune markers. Most of these studies, contrary to the previously mentioned body of studies, focused on the approach of observing the so called “preparatory immune response”, with the idea that a disgust reaction, i.e. the activation of BIS, causes an upregulation of the PIS. Among the immune markers most commonly used in these studies were IL-6, a cytokine produced by white blood cells and playing a key role in the inflammatory response; secretory IgA (S-IgA), a key component of mucosal immunity, serving as a non-inflammatory immunological defense (Mantis et al., 2011) or TNF-α, a potent pro-inflammatory cytokine produced during a microbial attack. In line with the proposed idea of a preparatory immune response, Schaller et al. (2010) showed an increase in levels of IL-6 after seeing photographs of infected people and Stevenson et al. (2011) observed an increase in participants’ levels of salivary TNF-α and albumin in response to disgusting stimuli. The findings regarding TNF-α and albumin were then replicated and supported in a later study (Stevenson et al., 2012), which additionally showed an increase in body temperature in response to disgusting stimuli. Furthermore, when separating disgust- and disease-related cues (by using a set of images that primarily evoked disgust and not disease concerns and vice-versa), a significant increase in levels of salivary TNF-α for both types of stimuli was observed in people who were generally more disgust sensitive (Stevenson et al., 2015). A recent study using three short videos representing different disease- and disgust-related stimuli (and one control) also showed a significant increase in S-IgA for all of the three salient stimuli (Keller et al., 2022). Interestingly, the authors also found that the levels of S-IgA increased less (the immune reaction was not as strong) in people who had higher contamination disgust, which could also support the existence of a compensatory relationship between BIS and PIS; i.e. the people whose immune system is less active in response to disgust or disease-relevant stimuli, could have elevated contamination disgust as a compensation (Keller et al., 2022).

A different approach to testing the relationship between PIS and BIS, without trying to induce a response, was taken by Kaňková et al. (2022), who assessed the activity of these systems separately. In a sample of women in their first trimester of pregnancy, the authors showed that lower levels of certain cytokines (FGF basic, Eotaxin, IFN-γ, IL-1β, IL-2, IL-4, IL-7, IL-17A, G-CSF, MCP-1, MIP-1α, PDGF-BB, IP-10, RANTES, and TNF-α) were associated with elevated disgust sensitivity, suggesting that when the immune system was not active enough, BIS activity was elevated to compensate for that (Kaňková et al., 2022). However, it needs to be noted that pregnancy is an immune specific period and the observed relationship may differ in non-pregnant population.

The relationship between the PIS and BIS is very complicated, with many different aspects. In our study, we aimed to add to the body of research regarding the relationship between these systems. Following the approach of Kaňková et al. (2022), we did not induce disgust to observe the physiological reaction, but instead examined the relationship in its existing state. Our goal was to replicate their findings in a non-pregnant population and thus assess whether their results can be generalized regardless of reproductive status or whether they involve specific mechanisms linked to pregnancy. As in the replicated study, we expected to observe evidence for a compensatory mechanism, where a less active PIS (represented by cytokine levels) would be associated with a more active BIS (represented by disgust sensitivity or avoidant behavior). Besides the aim to replicate these results using the same disgust questionnaire – the Disgust Scale-Revised (DS-R; Olatunji et al., 2007), we also chose to include another frequently used disgust questionnaire – the Three Domains of Disgust Scale (TDDS; Tybur et al., 2009). The TDDS includes, besides a pathogen-focused domain, also a sexual disgust domain, which may show some overlap in the associations we expect to observe with the pathogen domain. It also includes a conceptually separate moral domain, which we here expect to serve as a control measure, as we don't expect it to change in the context of the BIS/PIS relationship, and will allow us to separate pathogen-domain-specific effects. To address the limitations of text questionnaires we also decided to use tactile stimuli to evoke and measure disgust sensitivity, using the Tactile Disgust Stimuli Set (TDSS; Dlouhá and Kaňková, 2025). By using sensory stimuli (such as visual, olfactory and tactile) the potential pathogen threat is represented more directly to the participants, without the need to imagine the scenario. Additionally, to broaden our findings, we also included the Perceived Vulnerability to Disease questionnaire (PVD; Duncan et al., 2009), which reflects individuals' own perceptions about their immune state, which can modulate BIS activity, as well as their protective behaviors, which are the final (behavioral) component of the BIS, following the affective (disgust) reaction.

## 2 Methods

### 2.1 Data collection procedure

The participants were recruited as a part of a series of data collection for a larger project focused on the effect of internal and external factors on individual's performative, physiological, and psychological characteristics, involving blood sampling (see 2.3. Blood sampling and serum analysis). After going through blood sampling, the participants were asked whether they wanted to take part in an additional project focused on emotion and sensory perception (Dlouhá and Kaňková, 2025). They were then given printed study information and an informed consent to sign, including consent with using their blood serum (already collected) for further analysis. The additional testing took part in a separate room or section right away, with the main researcher being present and giving out instructions at all data collection points. The testing involved multiple activities, including first a tactile experimental task using the TDSS and then filling out a set of questionnaires, asking about basic sociodemographic and overall health questions, about the participants' disgust sensitivity and perceived vulnerability to disease (for more details, see 2.4. Measures).

The data was collected in three separate time points throughout one year – in April 2022 in Olomouc, Czech Republic (S1), and in June and October 2022 in Prague, Czech Republic (S2 and S3 respectively). This research project has been approved by the Institutional Review Board of the Faculty of Science, Charles University (Approval no. 2020/27) and conducted in accordance with the Declaration of Helsinki.

### 2.2 Sample characteristic

We wanted to confirm the results of the study by Kaňková et al. (2022), performed on a sample of pregnant women, on a population of non-pregnant women. Therefore, we have kept the same sample size (the sample size was determined by the size of the cytokine analysis kit) and similar inclusion criteria. In total, 78 healthy (without any serious chronic or autoimmune disorders) adult women were recruited for this study. Other inclusion criteria for recruitment were that the women were not pregnant, fluent in Czech, they filled out all or most of the questionnaires (primarily those focused on disgust sensitivity), underwent a successful blood sampling and were younger than 45. The sample characteristics are shown in Table 1.

**Table 1:** Sample characteristics for the whole sample, as well as for the three data collection times.

		Overall sample	S1	S2	S3
**N**		78	19	30	29
**Age (years; mean ± SD) range**		24.4 ± 5.61 19–42	25.6 ± 4.73 19–38	26.9 ± 6.69 19–42	21.1 ± 2.64 19–34
Education	High school	45 (59.2%)	3	15	27
	Post-secondary	3 (3.9%)	1	1	1
	University	28 (36.8%)	15	12	1
	missing	2	0	2	0
Place of residence	< 1,000	8 (10.7%)	0	3	5
	1,000–5,000	7 (9.3%)	3	0	4
	5,000–50,000	18 (24.0%)	3	8	7
	50,000–500,000	13 (17.3%)	9	1	3
	> 500,000	29 (38.7%)	2	17	10
	missing	3	2	1	0

### 2.3 Blood sampling and serum analysis

The blood sampling was performed by a certified healthcare professional, with experience drawing blood. Following blood collection, the methodology used was the same as in the replicated study. The blood sample was processed within 72 h of collection. Serum was obtained from the blood sample and stored at −20 °C until analyzed. The laboratory analyses of the serum samples were carried out at the Institute of Endocrinology in Prague in April 2023.

The blood serum samples were analyzed for cytokine concentrations using multiplex bead-based suspension array system (xMAP technology, Luminex Corp.) with the Bio-Plex Pro Human Cytokine 17-plex Assay (Cat. No: M5000031YV, Bio-Rad) under standard protocol. The cytokines detected with this assay are: G-CSF, GM-CSF, IFN-γ, IL-1β, IL-2, IL-4, IL-5, IL-6, IL-7, IL-8, IL-10, IL-12 (p70), IL-13, IL-17A, MCP-1 (MCAF), MIP-1β, TNF-α. All samples were run on a single plate. Data were evaluated by 5-parameter logistic regression with Bio-Plex Manager v. 6.1.1 (Bio-Rad).

### 2.4 Measures

#### 2.4.1 Disgust Scale-Revised

The DS-R (Olatunji et al., 2007) is a self-report questionnaire measuring a person's disgust sensitivity. It contains 25 items, in the form of statements, that the participants rate on a scale from 0 to 4, either depending on how much they agree with them (13 items) or how disgusting they find them (12 items). Higher score indicates higher disgust sensitivity. The questionnaire items are divided into three subscales: the core subscale (with 12 items concerned with disgust elicited by food, animals or bodily fluids), the animal reminder subscale (including 8 items related to injuries, mutilations, blood and death) and contamination subscale (with 5 items focused on interpersonal transmission of pathogens). This study used the Czech version of this questionnaire, by Polák et al. (2019). In case an answer to an item was missing from the questionnaire, the mean score of the corresponding subscale was substituted in its place. This was only done if less than one-fifth of the responses were missing from that subscale. The internal consistency for this questionnaire and its subscales, indicated by the Cronbach's α value, was 0.819 for the overall score, 0.660, 0.811 and 0.416 for the core, animal reminder and contamination subscales respectively. The reliabilities or the overall score and animal reminder are considered as good, but questionable for the core subscale and very poor/unacceptable for the contamination subscale. Caution is advised for data interpretation and further testing is necessary.

#### 2.4.2 Three Domains of Disgust Scale

The TDDS (Tybur et al., 2009) is another self-report tool used to measure individual disgust sensitivity. It consists of 21 items – statements, which participants rate on a scale from 0 to 6 depending on how disgusting they find them. Higher score indicates higher disgust sensitivity. The items in this questionnaire are divided into three domains: the pathogen, sexual and moral domain, each containing 7 items. In case an answer to an item was missing from this questionnaire, the mean score of its domain was substituted in its place. This was only done if less than one-fifth of the responses were missing for that particular domain. The internal consistency for this questionnaire and the three domains, indicated by the Cronbach's α value, was 0.608, 0.797 and 0.873 for the pathogen, sexual and moral domains respectively. These values are considered as good for the moral domain, acceptable for the sexual domain and questionable for the pathogen domain.

#### 2.4.3 Perceived vulnerability to disease

The PVD (Duncan et al., 2009) is used to measure an individual's perception of their own susceptibility to illness and their protective behaviors. Participants rate, on a scale from 0 to 6, how much they agree with 15 given statements. Out of these, 7 items belong to the Perceived Infectability subscale, and 8 items belong to the Germ Aversion subscale. As before, if a response to an item was missing, the mean score of its corresponding subscale was substituted. This was done only if one-fifth or less of the responses for that subscale were missing. The internal consistency, indicated by the Cronbach's α value, was 0.893 and 0.508 for the Perceived Infectability and Germ Aversion subscale respectively. The consistency value for the Perceived Infectability is considered as good and for the Germ Aversion as poor/unacceptable.

#### 2.4.4 Tactile Disgust Stimuli Set

The TDSS (Dlouhá and Kaňková, 2025) consists of 6 objects, meant to evoke disgust, hidden in boxes. These objects can be considered disgusting based on their tactile characteristics, while also being potentially reminiscent of specific disgusting objects or substances. The objects in this set are: soaked tapioca (fish insides), molasses on baking paper with dried fruits (flypaper with insects), bathroom tile with shaving cream (dirty bathroom), oatmeal with pear pieces (vomit), wet Play-Doh in excrement shape (excrement) and mayonnaise (pus). Participants are asked to put their hand inside, touch the object and answer a series of questions, including: “How disgusting did you find touching this object?”, “How disgusting would it be to put this object in your mouth?”, and “If you ate/licked this object, what is the probability that you would get ill?”. Responses are given on a scale from 1 (not at all/none) to 7 (very/high). Based on these responses, three scores are calculated as the sum of responses to each question from all objects: the Tactile Disgust score, Oral Disgust score, and Sickness Probability score respectively. The internal consistency, indicated by the Cronbach's α value, was 0.612 for the Tactile Disgust score, 0.703 for the Oral Disgust score and 0.771 for the Sickness Probability score.

### 2.5 Statistical analyses

The data was analyzed using the jamovi software (The jamovi project, 2022) and the SIMCA v. 12.1.1.1. (Umetrics, Umea, Sweden). Firstly, using a partial Pearson correlation analysis controlled for age (or a non-parametric equivalent in case of non-normal distribution), the scores obtained from the DS-R, TDDS and PVD questionnaires were correlated with each other to show the relationships between the different measures. The results of the Shapiro-Wilk normality tests for these measures are included in the Supplementary material (Table S1).

For the cytokine analyses we used the measured observed concentrations for each cytokine. In case more than half of the measurements were out of range, that particular cytokine was excluded from analysis. Overall, three cytokines (GM-CSF, IFN-γ, IL-4) were excluded and 14 cytokines (G-CSF, IL-1β, IL-2, IL-5, IL-6, IL-7, IL-8, IL-10, IL-12 (p70), IL-13, IL-17A, MCP-1 (MCAF), MIP-1β, TNF-α) were included in analyses. Descriptives of cytokine concentrations and more details on the function of the analyzed cytokines are included in the Supplementary material (Table S2 and section S1. *The function of the analyzed cytokines*, respectively).

As this study was largely a replication of the study by Kaňková et al. (2022), we performed the same type of analyses on our dataset. First, as primary analysis, to determine the associations between cytokine levels and the scores of DS-R (as done previously) and TDDS, TDSS and PVD (newly in our study), we used partial Kendall correlation with age as a covariate. We then adjusted the resulting p-values for multiple comparisons using the Benjamini–Hochberg procedure, with the false discovery rate set at 0.10. Based on a sensitivity analysis and the sample size for specific cytokines (N ranging from 42 to 78), the associations of approximately Kendall's tau b = 0.20–0.30 could be detected with 80% power.

As the main analysis, we conducted a multivariate regression with a reduction of dimensionality – orthogonal projections to latent structures (OPLS) (Trygg and Wold, 2002) – in order to further assess associations between cytokine levels, and those questionnaires, whose subscales showed significant associations in the primary analysis (DS-R and TDDS). The cytokine levels in these models served as the predictors and the disgust measure as the dependent variable. The OPLS model was then used to find the best linear combination of the independent variables (predictors) for an optimal estimation of the dependent variables. We included age as a covariate in the models. Variables were transformed using Box-Cox or Yeo-Johnson procedures (STATGRAPHICS Centurion 18 software (The Plains, VA, USA)), and cytokines with right-skewed distributions were log-transformed. Transformations were applied once per variable to stabilize variance prior to OPLS modelling.

The data are available at: https://doi.org/10.6084/m9.figshare.29796332.v2.

## 3 Results

### 3.1 Intercorrelation of psychometric measures

A partial Pearson correlation analysis revealed moderate positive intercorrelations between two of the DS-R subscales and the pathogen domain of TDDS. The core and contamination subscales of DS-R (as well as the overall DS-R score) and the pathogen and moral domains of TDDS were also significantly positively correlated with the Germ Aversion subscale of PVD. The sexual domain of TDDS weakly correlated with the pathogen domain of TDDS. The Perceived Infectability subscale of PVD did not significantly correlate with any other measures. For more details, see Table 2.

**Table 2:** Pearson/Spearman correlation analysis between all questionnaire measures.

		DS-R				TDDS			PVD
		Overall	Core	Animal Reminder	Contamination	Pathogen	Sexual	Moral	Perceived Infectability
DS-R	Core	0.860***	-	-	-	-	-	-	-
	Animal reminder	0.824***	0.491***	-	-	-	-	-	-
	Contamination	0.627***	0.434***	0.328**	-	-	-	-	-
TDDS	Pathogen	0.347**	0.369**	0.190	0.268*	-	-	-	-
	Sexual	0.129^†^	0.103^†^	0.048^†^	0.209^†^	0.271*^†^	-	-	-
	Moral	0.027	−0.047	0.013	0.193	0.007	0.206^†^	-	-
PVD	Perceived Infectability	0.072^†^	−0.112^†^	−0.032^†^	0.123^†^	0.129^†^	−0.203^†^	−0.199^†^	-
	Germ Aversion	0.304**	0.229*	0.148	0.487***	0.297**	0.067^†^	0.250*	0.173^†^

Pearson's r or Spearman's rho (those analyses are marked with^†^) values are reported in the table, with significance marked as follows: *<0.05, **<0.01 and ***<0.001. Analyses are controlled for age.

### 3.2 Preliminary analysis

To assess associations between the two disgust measures, DS-R and TDDS, and cytokine levels, we first used partial Kendall correlation controlled for age. The results showed significant positive associations between the DS-R core subscale and IL-5 and IL-12, between the DS-R overall score and IL-5 and IL-12 and between the TDDS pathogen domain and MIP-1β. We also observed a negative correlation between the DS-R core subscale and MCP-1, the DS-R contamination subscale and IL-1β and TNF-α, and between the TDDS moral domain and MIP-1β. None of these results withstood the Benjamini-Hochberg correction for multiple testing. For more details on this analysis, see Table 3. We have also performed a residual-based partial Bayesian Kendall's correlation on log-transformed data, and the results of this analysis are included in the Supplementary material (Table S3).

**Table 3:** Partial Kendall correlation between cytokine levels, DS-R and TDDS.

		DS-R				TDDS		
		Core	Animal Reminder	Contamination	Overall Score	Pathogen	Sexual	Moral
G-CSF	tau b	−0.008	−0.026	−0.086	−0.042	0.005	0.066	−0.024
	p	0.930	0.772	0.330	0.637	0.959	0.455	0.786
	N	60	61	61	60	61	61	60
IL-1β	tau b	−0.021	−0.042	**−0.177**	−0.051	0.009	0.017	−0.099
	p	0.793	0.603	**0.027**	0.523	0.911	0.836	0.218
	N	73	74	74	73	74	74	73
IL-2	tau b	0.120	0.047	−0.022	0.091	0.045	0.044	−0.047
	p	0.175	0.595	0.807	0.302	0.612	0.623	0.597
	N	61	61	61	61	61	61	60
IL-5	tau b	**0.199**	0.072	0.123	**0.179**	0.087	0.021	−0.032
	p	**0.025**	0.411	0.163	**0.043**	0.324	0.814	0.718
	N	61	62	62	61	62	62	62
IL-6	tau b	0.115	0.012	−0.056	0.058	0.041	−0.062	−0.140
	p	0.157	0.879	0.485	0.475	0.612	0.440	0.085
	N	72	73	73	72	73	73	72
IL-7	tau b	−0.022	−0.061	−0.062	−0.020	−0.044	0.007	−0.039
	p	0.789	0.449	0.447	0.711	0.583	0.930	0.635
	N	71	72	72	71	72	72	71
IL-8	tau b	0.028	0.033	−0.035	0.029	0.073	0.023	−0.092
	p	0.716	0.668	0.655	0.707	0.348	0.772	0.239
	N	77	78	78	77	78	78	77
IL-10	tau b	0.144	−0.051	−0.022	0.026	−0.026	−0.079	0.098
	p	0.183	0.637	0.834	0.814	0.811	0.463	0.368
	N	42	43	43	42	43	43	42
IL-12 (p70)	tau b	**0.193**	0.066	0.071	**0.167**	0.043	−0.028	0.009
	p	**0.013**	0.398	0.361	**0.033**	0.578	0.718	0.908
	N	77	78	78	77	78	78	77
IL-13	tau b	0.042	0.068	0.014	0.051	−0.067	0.141	−0.043
	p	0.604	0.391	0.858	0.524	0.403	0.077	0.595
	N	73	74	74	73	74	74	73
IL-17A	tau b	0.025	−0.074	−0.023	−0.005	−0.075	−0.006	−0.107
	p	0.762	0.363	0.780	0.953	0.353	0.939	0.188
	N	71	72	72	71	72	72	71
MCP-1 (MCAF)	tau b	**−0.159**	−0.067	−0.057	−0.139	−0.104	−0.007	−0.007
	p	**0.042**	0.389	0.463	0.075	0.182	0.931	0.925
	N	77	78	78	77	78	78	77
MIP-1β	tau b	0.082	0.047	0.005	0.094	**0.153**	0.043	**−0.166**
	p	0.296	0.545	0.946	0.229	**0.049**	0.577	**0.034**
	N	77	78	78	77	78	78	77
TNF-α	tau b	0.015	−0.050	**−0.164**	−0.063	−0.019	0.085	−0.089
	p	0.843	0.519	**0.035**	0.419	0.802	0.274	0.256
	N	77	78	**78**	77	78	78	77

Controlled for age.

We also performed Kendall correlation analyses between cytokine levels and scores of the PVD questionnaire and the TDSS. Neither measure was significantly associated with any cytokine levels. For more details on these analyses, see Table 4.

**Table 4:** Partial Kendall correlation between cytokine levels, PVD and TDSS.

		PVD		TDSS		
		Perceived Infectability	Germ Aversion	Tactile disgust	Oral disgust	Sickness probability
**G-CSF**	tau b	−0.138	−0.028	0.059	−0.089	0.027
	p	0.076	0.721	0.445	0.248	0.729
	N	61	60	61	61	61
**IL-1β**	tau b	−0.013	−0.141	−0.051	−0.074	−0.124
	p	0.868	0.072	0.512	0.336	0.107
	N	74	73	74	74	74
**IL-2**	tau b	−0.073	0.028	0.080	0.001	−0.011
	p	0.349	0.724	0.298	0.984	0.892
	N	61	60	61	61	61
**IL-5**	tau b	0.027	0.134	0.032	0.040	0.035
	p	0.724	0.086	0.680	0.601	0.653
	N	62	61	62	62	62
**IL-6**	tau b	0.066	0.008	0.032	−0.037	−0.084
	p	0.396	0.920	0.679	0.634	0.274
	N	73	72	73	73	73
**IL-7**	tau b	0.002	−0.037	0.046	−0.043	−0.001
	p	0.977	0.632	0.550	0.573	0.988
	N	72	71	72	72	72
**IL-8**	tau b	−0.043	−0.103	0.040	0.066	−0.031
	p	0.578	0.188	0.603	0.393	0.685
	N	78	77	78	78	78
**IL-10**	tau b	−0.017	−0.015	0.016	−0.016	−0.037
	p	0.832	0.849	0.840	0.836	0.629
	N	43	43	43	43	43
**IL-12 (p70)**	tau b	0.008	0.084	−0.088	0.022	0.037
	p	0.916	0.283	0.255	0.776	0.628
	N	74	73	78	78	78
**IL-13**	tau b	−0.097	−0.076	0.047	0.029	0.005
	p	0.211	0.332	0.542	0.705	0.945
	N	72	71	74	74	74
**IL-17A**	tau b	−0.140	0.004	−0.076	−0.057	−0.150
	p	0.071	0.954	0.324	0.457	0.051
	N	78	77	72	72	72
**MCP-1 (MCAF)**	tau b	0.047	−0.013	−0.034	−0.022	−0.112
	p	0.545	0.868	0.659	0.771	0.146
	N	78	77	78	78	78
**MIP-1β**	tau b	−0.034	−0.027	−0.016	0.002	−0.040
	p	0.663	0.732	0.839	0.979	0.603
	N	78	77	78	78	78
**TNF-α**	tau b	−0.057	−0.046	−0.060	−0.016	−0.038
	p	0.462	0.554	0.441	0.840	0.623
	N	78	77	78	78	78

Controlled for age.

### 3.3 Main analyses

We used the multivariate OPLS models for the main analyses of associations between the DS-R subscales, the DS-R overall score or TDDS domains (matrix Y) and cytokine levels and age (matrix X). One model was constructed for each subscale/domain or the overall DS-R score separately. Because we found no significant correlations between cytokine levels and PVD, nor the tactile experimental variables, we constructed no further OPLS models involving these measures.

The model for DS-R core subscale showed that disgust was significantly predicted by IL-2, IL-5, IL-10, IL-12, specifically that higher levels of these cytokines predicted higher levels of core disgust. Additionally, MCP-1 was also a significant predictor, but in the opposite direction, such that lower levels of MCP-1 predicted higher core disgust. This model explained 18.4% of variability (14% after cross-validation) of the total variability in DS-R core disgust.

For the DS-R contamination subscale, the model showed that contamination disgust was significantly predicted by IL-1β, IL-2, IL-6, IL-8 and TNF-α; lower levels of these cytokines predicted higher contamination disgust. The model explained 9.7% of variability (6.3% after cross-validation) in contamination disgust of the DS-R.

For the animal reminder subscale of the DS-R, the model showed no significant associations.

In the model for the DS-R overall score, disgust was significantly predicted by IL-2, IL-5 and IL-12, such that higher levels of these cytokines predicted higher overall DS-R score. MCP-1 was also a significant predictor – lower levels of MCP-1 predicted higher overall DS-R score. This model explained 18.3% of variability (14% after cross-validation) of the overall DS-R score variability. All models for the DS-R are presented in Table 5.

**Table 5:** Associations between disgust measured using DS-R (core subscale/contamination subscales/overall score) and cytokine levels evaluated by OPLS model and multiple regression.

	Variable	OPLS, predictive component			Multiple regression	
		Component loading	t-statistics	R^*a*^	Regression coefficient	t-statistics
Relevant predictors (matrix X)	IL-2	0.403	5.07	0.586**	0.082	1.64
	IL-5	0.613	13.91	0.867**	0.191	8.25**
	IL-10	0.379	4.88	0.561**	0.093	4.99**
	IL-12	0.479	5.11	0.662**	0.125	2.32*
	MCP-1	−0.380	−2.40	−0.525*	−0.168	−2.16*
(matrix Y)	**DS-R Core**	1.000	2.93	0.428*		
Explained variability		18.4% (14% after cross-validation)				

Relevant predictors (matrix X)	IL-1β	−0.560	−8.86	−0.876**	−0.130	−2.70*
	IL-2	−0.294	−5.07	−0.474**	−0.051	−1.55
	IL-6	−0.452	−5.88	−0.716**	−0.084	−2.16*
	IL-8	−0.389	−6.95	−0.607**	−0.057	−1.84
	TNF-α	−0.515	−11.17	−0.801**	−0.102	−2.87*
(matrix Y)	**DS-R Contamination**	1.000	2.78	0.311*		
Explained variability		9.7% (6.3% after cross-validation)				

Relevant predictors (matrix X)	IL-2	0.354	3.51	0.467**	0.060	2.10*
	IL-5	0.631	11.35	0.791**	0.179	3.79**
	IL-12	0.495	3.69	0.614**	0.156	6.80**
	MCP-1	−0.548	−3.00	−0.679**	−0.239	−2.12*
(matrix Y)	**DS-R Overall Score**	1.000	2.58	0.428*		
Explained variability		18.3% (14% after cross-validation)				

^a^R … Component loadings expressed as correlation coefficients with predictive component, *p < 0.05, **p < 0.01.

Regarding the TDDS, the models for the pathogen and sexual domains showed no significant associations with cytokine levels or age. For the moral domain, however, we found that IL-1β, IL-2, IL-5, IL-12, IL-17A, MIP-1β and TNF-α were significant predictors for this model. Specifically, lower levels of these cytokines predicted higher disgust in the moral domain of TDDS. This model explained 11.6% of variability (8.8% after cross-validation) of the TDDS moral domain disgust. The model is displayed in Table 6.

**Table 6:** Associations between TDDS moral domain and cytokine levels evaluated by OPLS model and multiple regression.

	Variable	OPLS, predictive component			Multiple regression		
		Component loading	t-statistics	R^*a*^	Regression coefficient		t-statistics
Relevant predictors (matrix X)	IL-1β	−0.397	−11.54	−0.731**	−0.056		−2.32*
	IL-2	−0.346	−13.73	−0.701**	−0.096		−3.21**
	IL-5	−0.334	−11.51	−0.724**	−0.084		−2.06*
	IL-12	−0.432	−7.95	−0.768**	−0.063		−2.04*
	IL-17A	−0.440	−11.60	−0.812**	−0.114		−3.13**
	MIP-1β	−0.306	−5.40	−0.544**	−0.053		−1.85
	TNF-α	−0.447	−9.69	−0.800**	−0.096		−1.77
(matrix Y)	**TDDS moral**	1.000	2.54	0.348*			
Explained variability		11.6% (8.8% after cross-validation)					

^a^R … Component loadings expressed as correlation coefficients with predictive component, *p < 0.05, **p < 0.01.

## 4 Discussion

In our study, we aimed to investigate the relationship between the PIS, represented here by cytokine levels, and the BIS, represented by text-based and experiment-based disgust scores, as well as PVD questionnaire scores. We aimed to investigate whether such association exists in a non-pregnant population sample and therefore we only propose possible explanations or potential roles of other factors as pathways for future research. The study partly replicated previous research on pregnant women (Kaňková et al., 2022), specifically the assessment of the BIS using the DS-R questionnaire. Based on this study and other previous research (see the Introduction for more detail), we primarily expected to observe a negative relationship between disgust and selected cytokine levels. The results of our study, however, were twofold. On one hand, in line with the study on pregnant women, we found negative relationship between the contamination subscale of the DS-R and IL-1β and IL-2, as well as between both, the core subscale of the DS-R and the overall score and MCP-1. Additionally, we observed a negative relationship between disgust sensitivity on the contamination subscale and IL-6, IL-8 and TNF-α, which were not observed in the previous study. On the other hand, we also observed a positive relationship between core disgust sensitivity and IL-2, IL-5, IL-10 and IL-12, and the overall DS-R score and IL-2, IL-5 and IL-12. We also showed a significant negative association between the moral disgust domain of the TDDS, which has not been previously studied in this context, and the following cytokines: IL-1β, IL-2, IL-5, IL-12, IL-17A, MIP-1β and TNF-α. We found no significant correlations between cytokine levels and the PVD, nor the tactile experimental variables. However, our results should not be over-generalized and should be treated with caution, especially regarding those types of disgust measured by subscales that show low reliability (such as the contamination subscale). The findings involving the overall DS-R score, which shows good reliability are therefore those that should be considered the most credible.

In line with the study on pregnant women (Kaňková et al., 2022), we confirmed negative associations between levels of pro-inflammatory cytokines IL-1β and IL-2 and contamination disgust and MCP-1 and core and overall disgust. We also found a negative association of pro-inflammatory cytokines TNF-α, IL-8 and IL-6 with contamination disgust. It needs to be noted that the associations of IL-2 and IL-8 were observed only in the OPLS model and did not remain significant in the multiple regression analysis, suggesting that their relationship with contamination disgust may reflect shared variance with other cytokines rather than independent effects. The IL-1β, IL-6 and TNF-α are often used as general markers of inflammation, which is also why they are most frequently used in studies monitoring immune response to BIS activation (see Introduction). On the other hand, the MCP-1 and IL-8 are chemokines and more involved in signaling and attracting immune cells to specific infection sites (Kany et al., 2019) and IL-2 is responsible for T-cell differentiation and regular maintenance (Boyman and Sprent, 2012). These findings may support the hypothesis of a compensatory mechanism between BIS and PIS – if the PIS is inactive or insufficiently activated, an increase of BIS activity will compensate for that, so the individual remains protected. On the other hand, with our analyses, we are not able to determine causality. Therefore, this association may also be explained simply by an active and effective BIS – lower levels of cytokines may also signal that no immune-relevant event has taken place, due to higher levels of disgust providing sufficient behavioral protection. Regarding the nature of the association between those cytokines and contamination disgust, a similar pattern was also reported by Keller et al. (2022) who observed a negative relationship between contamination disgust and changes in a different immune marker – S-IgA levels, such that people whose immune reaction, marked by change in S-IgA levels, was not as strong had higher contamination disgust. On the other hand, in contrast to our results, Stevenson et al. (2015) reported increased TNF-α levels in response to disgust- and disease-related cues among individuals with higher disgust sensitivity, suggesting a simultaneous activation of both BIS and PIS. Unlike our study, however, their design allowed them to test a more direct causal pathway, whereby heightened contamination disgust may shape immune activation rather than merely compensate for insufficient PIS activity. Moreover, it can be noted while Keller et al. (2022) used the DS-R to measure disgust sensitivity, Stevenson et al. (2015) observed their results for the pathogen domain of the TDDS, which did not show any significant negative association with any cytokines in our study. Lastly, it needs to be noted that many of these findings have been observed with the contamination subscale and therefore, due to its low reliability, should be treated with caution.

While some of the results align with those reported by Kaňková et al. (2022), several cytokines, namely IFN-γ, IL-4, IL-7 and IL-17A, showed a significant negative association with disgust in their sample of pregnant women, but not in our sample of non-pregnant women. We did show an association of IL-17A with the moral domain of TDDS, but as that is a specific type of disgust, unrelated to pathogen concerns, it is discussed separately below. This discrepancy highlights the importance of considering the unique immunological context of pregnancy. During pregnancy, the maternal immune system undergoes numerous changes and modifications (Mor et al., 2011; Racicot et al., 2014), cytokine levels can be present in different concentrations and the dynamic between PIS and BIS can change during this time. Indeed a study has shown that levels of IFN-γ differed significantly between non-pregnant and pregnant women (Vassiliadis et al., 1998). A different longitudinal study showed dynamic changes in cytokine levels throughout the whole pregnancy, with levels of some pro-inflammatory cytokines being highest in first and third trimester (Jarmund et al., 2021). Levels of many cytokines (such as IL-17A) seem to increase from the first to third trimester (Stokkeland et al., 2019). This suggests that the compensatory mechanisms between PIS and BIS may become more pronounced during pregnancy, especially in the first trimester, serving to maintain protection against pathogens that could compromise fetal development.

We have also observed a positive association between disgust and cytokine levels, specifically between core disgust, overall DS-R and IL-2, IL-5, and IL-12 and between IL-10 and core disgust. IL-5 is expressed in eosinophils, which play a key role in the pulmonary inflammation associated with allergies (Greenfeder et al., 2001; Pelaia et al., 2019). Elevated IL-10 levels have then been associated with IL-5-related Th2 activity in asthma, influencing IL-5 production (Yang et al., 2000) and the IL-10/IL-5 balance has been shown to be linked to patient's current clinical state of asthma (Tomiita et al., 2015). IL-12 has also been shown to be higher in patients with asthma (Wong et al., 2001) and it played a role in allergic airway inflammation during the effector phase on an animal model (Meyts et al., 2006). Additionally, IL-2 has also been shown to play a role in the asthmatic response (Boonpiyathad et al., 2013). Besides their role discussed above, the cytokines IL-5, IL-12 and IL-10 have also been shown to be associated with some aspects of mental health. Specifically, IL-5 and IL-12 have been associated with neuroticism and depression (Nahar et al., 2023; Schmidt et al., 2018) and with prenatal depressive symptoms in pregnant women (Karlsson et al., 2017). Additionally, IL-10 was shown to be associated with negative mood in pregnancy (Takács et al., 2024) and with severe anxiety (Leff Gelman et al., 2019).

Based on the findings of these studies, we may further speculate on the reasons behind our observed results. There exists an area of research that focuses on the connection between mental health and respiratory problems – studies have shown a significant positive association between the prevalence of anxiety and asthma (Del Giacco et al., 2016; Katon et al., 2004). Additionally, disgust is often positively associated with mental health, such as anxiety disorders (Cervin et al., 2020; Knowles et al., 2018; Stasik-O’Brien et al., 2021), depression (Ille et al., 2014), and general neuroticism traits (Olatunji et al., 2008). Overall, there seems to be some overlap between these research areas. The observed positive associations between disgust and cytokines could therefore, in theory, be related to the changes in mental health and even tied to chronic respiratory issues. However, our results are only exploratory in nature and as such, our results and their interpretations are only suggestions for potential areas of future interest, possibly serving as a starting point for more focused research in clinical groups.

Interestingly, we found no significant association between cytokine levels and pathogen avoidance motivation measured by the PVD. These findings are consistent with some of the previous studies that also observed primarily associations between disgust and immune markers, but did not find significant links with the PVD (Keller et al., 2022; Stevenson et al., 2015). On the other hand, a study by Gassen et al. (2018), which focused specially on pathogen avoidance motivation (measured by the Germ Aversion subscale of the PVD), showed that individuals with higher pathogen avoidance motivation had lower levels of plasma IL-6 measured in vivo and less spontaneous release of IL-1β, IL-6, and TNF-α in vitro. However, this higher pathogen avoidance did not predict a decreased release in response to immune stimulation, suggesting that increased BIS activity does not necessarily have to predict lower PIS activity. Moreover, the study showed that people with higher pathogen avoidance motivation also had lower oxidative stress. Their discussion of these results brings another possible explanation to the mechanisms between BIS and PIS – that higher BIS activity could protect the body from the harmful effects of inflammation and oxidative stress, while the activity of PIS in times of infection would remain normal. Although our results did not show any significant negative relation between pathogen avoidance and cytokine levels, which would be in line with this explanation, we did observe this relationship between disgust and levels of IL-1β and TNF-α. This could suggest that while pathogen avoidance and disgust are both different components of the BIS, they may represent different mechanisms of behavioral immunity. It is worth noting that the study by Gassen et al. (2018) included a much younger (student) sample and over half of the participants were men. It is possible that different behavioral strategies are differently represented in men and women and in different age groups.

We also didn't find any associations between the variables related to tactile disgust and cytokine levels. In the original study establishing the TDSS tactile measure (Dlouhá and Kaňková, 2025), which had an overlapping sample with the current study, the strongest association was with the animal reminder subscale of the DS-R. This subscale also turned out to play a significant role in how the different tactile objects were rated (along with the Germ Aversion subscale of the PVD). The authors discuss this via the connection between body vulnerability and body envelope concerns (represented by the animal reminder disgust) and the proximate modality of touch. In the current study, no cytokine levels were associated with animal reminder disgust. Overall, it seems that tactile disgust is, similarly to the pathogen avoidance motivation mentioned above, connected to a different component of the BIS. Therefore, tactile disgust measures might not be the most suitable for exploring the relationship between BIS and PIS. Measures using other sensory modalities, such as smell, which is more involved in pathogen detection from the environment than touch, could be a better alternative to use in future studies.

Regarding the TDDS, our results did not show any statistically significant association between cytokine levels and the pathogen or sexual domains of this questionnaire. While the pathogen domain, in theory, overlaps with the core and contamination disgust of the DS-R, there are several studies that have shown stronger effects for the DS-R than the TDDS when using both questionnaires (e.g. Dlouhá et al., 2024; Dlouhá and Kaňková, 2025), suggesting perhaps higher sensitivity of the former measure. Considering sexual disgust specifically, it also needs to be noted that while it does partially serve a pathogen-protective role (mainly against sexually transmitted diseases) its main function is to protect from sexual behavior that could lead to lower reproductive success (Tybur et al., 2009).

On the other hand, we observed significant negative associations between several pro-inflammatory cytokines (IL-1β, IL-2, IL-5, IL-12, IL-17A, MIP-1β and TNF-α) and the moral disgust domain of the TDDS. To our knowledge, no studies have yet directly focused on the link between moral disgust and PIS activity and this finding should therefore be treated as exploratory. While they may be some overlap in scenarios that affect both moral and pathogen disgust, such as the Covid-19 pandemic, which, at the time, provided context that was both socially and disease-relevant, as illustrated by Schwarz et al. (2023), these two concepts of disgust are quite different. It needs to be considered that the negative associations of this particular domain might not be related to the previously discussed compensatory mechanism, i.e. that people with lower levels of these cytokines have elevated moral disgust. Rather, it is possible that people with elevated levels of these cytokines, suggesting an ongoing immune activation, have lower levels of moral disgust. As moral disgust is concerned with violations affecting the social group, it is possible that people whose immune systems are currently active focus more on managing their own health than on the behavior of others. A review showed that people who were more lonely or isolated had increased pro-inflammatory activity (Eisenberger et al., 2017), however, loneliness does not necessarily indicate less concern about social norms. It also needs to be said that results in this area of research generally suggest the opposite – that people concerned about their health pay more attention to social norms and violations (Schwambergová et al., 2023). Overall, the relationship between immune markers and moral disgust might require more detailed research that would include the observation of additional factors.

### 4.1 Strengths and limitations

The strengths of this study are the simultaneous use of multiple disgust questionnaires, as well as the use of a tactile experimental task to evoke and measure disgust. This allows us to better understand how different components of the BIS relate to markers of immunity. The use of a laboratory kit for the analysis of 17 cytokines allowed us to examine not only the expected associations, but also new relationships, taking into account different aspects of disgust. For future research, however, a use of two (or three) kits that would allow for duplicate (or triplicate) testing, would be optimal.

One limitation of the study may be the relatively healthy sample of women without serious medical conditions, which could have reduced variability in immune functioning and may have contributed to the lack of certain associations that might be detectable in a more immunologically diverse or immunosuppressed population. Our findings should not be used for generalization on a broader population, but rather serve as a starting point for future research.

Another limitation, possibly marginally related to immunity, is that we did not have information about the women's cycle phase. However, the aim of this study was to assess the current association between immune markers and disgust sensitivity, rather than to assess the causes of any observed immune changes in detail. This may become the subject of further research. While several studies (Miłkowska et al., 2019; Strzelczyk et al., 2026; Żelaźniewicz et al., 2016) have found positive associations between disgust and women's cycle phase, these findings are often small-scale or observed only on specific subsamples. Moreover, several studies have repeatedly observed no significant effect of cycle phase on disgust sensitivity (Dlouhá et al., 2024; Jones et al., 2018; Rafiee et al., 2022; Stern and Shiramizu, 2022; Timmers et al., 2018), not even in a study comparing pregnant and non-pregnant women (Dlouhá et al., 2024). Furthermore, in a study by Keller and Diekhof (2024), the authors showed no effect of cycle phase on S-IgA levels or disgust sensitivity.

Lastly, we cannot say with certainty that our results support the existence of a compensatory mechanism between BIS and PIS, as the participants were not in a controlled environment prior to their blood collection. We therefore cannot exclude that the observed associations were caused by a prior exposure to disgusting stimuli and a subsequent increase in cytokine levels.

## 5 Conclusion

This study adds to the body of research regarding the relationship between the physiological immune system and the behavioral immune system. Similarly to a previous study in pregnant women, our results provide some evidence for a possible compensatory-type relationship, as some cytokine levels were negatively associated with disgust sensitivity. However, as we are unable to determine the causality of the associations at this point, the relationship may also be signal an effective behavioral protection. Although our findings allow for some generalization from pregnant to non-pregnant populations, several cytokines that showed significant negative associations with disgust in pregnant women did not show the same pattern in our sample of non-pregnant women, further suggesting the unique immunological context of pregnancy. Moreover, we found positive associations between disgust sensitivity and cytokine levels that have repeatedly been shown to be associated with respiratory conditions, such as asthma or allergies, as well as to mental health, such as neuroticism or anxiety. This therefore opens up the possibility for future research investigating the relationship between disgust, mental health and respiratory functioning. In addition, we observed negative associations between some pro-inflammatory immune markers and moral disgust. Due to a lack of existing research in this area, we can only speculate on the underlying mechanism of this association, as it requires further study. Lastly, we investigated whether cytokine levels were associated with other aspects of the behavioral immune system, specifically the vulnerability to disease and pathogen avoidance motivation, as well as disgust in response to tactile stimuli. None of these measures showed any significant association with the observed cytokine levels. This highlights the complexity and heterogeneity of the mechanisms that constitute the behavioral immune system and disgust sensitivity itself, suggesting that different components of behavioral protection may be differently linked to physiological processes. As these results are based on a healthy population sample and the study has shown novel associations in this type of study, our findings may serve as a base point for future targeted research on clinical samples rather than having definite conclusions.

## CRediT authorship contribution statement

**Daniela Dlouhá:** Conceptualization, Data curation, Formal analysis, Investigation, Methodology, Project administration, Validation, Writing – original draft, Writing – review & editing. **Martin Hill:** Formal analysis, Investigation, Resources, Software, Validation, Writing – review & editing. **Josef Včelák:** Investigation, Resources, Supervision, Writing – review & editing. **Šárka Kaňková:** Conceptualization, Formal analysis, Funding acquisition, Investigation, Methodology, Resources, Supervision, Writing – original draft, Writing – review & editing.

## Ethics declaration

Written informed consent to take part in the study and to publish the article has been obtained from all participants or their legal representatives. The privacy rights of participants have been observed.

This study was performed in compliance with relevant laws, regulatory frameworks and guidelines where the research took place. This study was approved by the Institutional Review Board of the Faculty of Science, Charles University (Approval No. 2020/27).

## Data availability

The open data are available at: https://doi.org/10.6084/m9.figshare.29796332.v2.

## Funding

This project was supported by the Czech Science Foundation, project GA CR (23-05519S). The authors MH and JV were additionally funded by the Ministry of Health of the Czech Republic Institutional Support grant no. RVO 00023761 — Institute of Endocrinology.

## Declaration of competing interest

The authors declare that they have no known competing financial interests or personal relationships that could have appeared to influence the work reported in this paper.
