# Supplementary material for: The relationship between disgust sensitivity and serum cytokine levels in non-pregnant women: a generalization of previous results on pregnant population
Source: Brain Behav Immun Health. 2026 Sep 22;58:101374. doi: 10.1016/j.bbih.2026.101374 (PMC13672964; doi:10.1016/j.bbih.2026.101374)
Supplement: Supplementary file 1 [file mmc1.docx]

Table S1. Shapiro-Wilk tests for the psychometric measures

|  |  | N | Shapiro–Wilk W | p |
| --- | --- | --- | --- | --- |
| DS-R | Overall score | 77 | 0.985 | 0.531 |
|  | Core | 77 | 0.972 | 0.093 |
|  | Animal Reminder | 78 | 0.981 | 0.286 |
|  | Contamination | 78 | 0.979 | 0.227 |
| TDDS | Pathogen | 78 | 0.975 | 0.120 |
|  | Sexual | 78 | 0.955 | 0.008 |
|  | Moral | 77 | 0.970 | 0.068 |
| PVD | Perceived Infectability | 78 | 0.960 | 0.015 |
|  | Germ Aversion | 77 | 0.982 | 0.329 |

Table S2. Descriptives of analysed cytokine concentrations (in pg/ml)

|  | N | Mean | Median | SD | Minimum | Maximum |
| --- | --- | --- | --- | --- | --- | --- |
| G-CSF | 61 | 56.1 | 29.5 | 85.3 | 6.41 | 464 |
| IL-1β | 74 | 1.06 | 0.40 | 1.35 | 0.10 | 5.68 |
| IL-2 | 61 | 2.99 | 2.13 | 3.98 | 0.68 | 21.6 |
| IL-5 | 62 | 51.4 | 18.8 | 118 | 2.99 | 621 |
| IL-6 | 73 | 2.49 | 1.95 | 1.89 | 0.30 | 8.12 |
| IL-7 | 72 | 10.3 | 7.34 | 11.3 | 0.88 | 57.4 |
| IL-8 | 78 | 142 | 67.0 | 182 | 4.95 | 857 |
| IL-10 | 43 | 5.47 | 1.63 | 9.40 | 0.71 | 41.3 |
| IL-12 (p70) | 78 | 16.0 | 11.7 | 19.5 | 0.63 | 116 |
| IL-13 | 74 | 1.21 | 0.76 | 1.12 | 0.37 | 5.06 |
| IL-17A | 72 | 2.70 | 1.88 | 3.01 | 0.49 | 16.4 |
| MCP-1 (MCAF) | 78 | 49.4 | 41.6 | 33.6 | 11.5 | 187 |
| MIP-1β | 78 | 141 | 84.3 | 155 | 15.5 | 1 030 |
| TNF-α | 78 | 25.7 | 22.8 | 11.4 | 10.2 | 71.5 |

*S1. The function of the analysed cytokines*

**G-CSF** is essential for neutrophil production and maturation. Its levels increase during infection (Kurzrock, 2003).

**IL-1β** is a major pro-inflammatory cytokine and is a sign of an ongoing infection, inflammation on injury. It plays a part in triggering fever, activating lymphocytes and expression of the acute phase genes (Cameron & Kelvin, 2013).

**IL-2** is important in the process of T-cell proliferation and activation, while also having an effect on a number of other immune-relevant cells, such as B cells or NK cells (Cameron & Kelvin, 2013).

**IL-5** is expressed in eosinophils, which play a key role in the pulmonary inflammation associated with allergies (Greenfeder et al., 2001; Pelaia et al., 2019).

**IL-6** is a cytokine produced by white blood cells and playing a key role in the inflammatory response by being essential in the regulation of immune cell proliferation and differentiation (Garbers et al., 2018).

**IL-7** plays a role in maturation of the immune system by stimulating the development of immature B cells, thymocytes and mature T cells (Cameron & Kelvin, 2013).

**IL-8** attracts neutrophils from the bloodstream to the sites of infection, where it is able to activate them (Charles A Janeway et al., 2001).

**IL-10** is an anti-inflammatory cytokine by inhibiting the synthesis of a number of pro-inflammatory cytokines via macrophages or Th-1 cells (Cameron & Kelvin, 2013; Sabat et al., 2010). Elevated IL-10 levels have then been associated with IL-5-related Th2 activity in asthma, influencing IL-5 production (Yang et al., 2000) and the IL-10/IL-5 balance has been shown to be linked to patient’s current clinical state of asthma (Tomiita et al., 2015).

**IL-12 (p70)** promotes differentiation/activation of Th1 cells and NK cells, thus supporting cell-mediated immunity (Cameron & Kelvin, 2013).

**IL-13** plays a role in the allergic response by promoting the immunoglobulin E response, eosinophil activation and mucous production (Akdis et al., 2016).

**IL-17A** promotes the production of pro-inflammatory chemokines, hematopoietic cytokines and acute phase genes, but can often drive chronic inflammatory conditions (Gaffen, 2008; Harris & Patel, 2025)

**MCP-1 (MCAF)** attracts monocytes/macrophages to sites of infection and activates them (Charles A Janeway et al., 2001; Deshmane et al., 2009a).

**MIP-1β** is involved in the recruitment and activation of monocytes (Deshmane et al., 2009b)

**TNF-α** is a major pro-inflammatory cytokine. It induces local inflammatory response, can directly or indirectly induce fever and is able to induce apoptosis (Cameron & Kelvin, 2013; Charles A Janeway et al., 2001).

Table S3. Residual-based partial Bayesian Kendall correlation analysis between cytokine levels and disgust questionnaires (DS-R and TDDS)

|  | | DS-R | | | | TDDS | | |
| --- | --- | --- | --- | --- | --- | --- | --- | --- |
|  |  | Core | Animal Reminder | Contamination | Overall Score | Pathogen | Sexual | Moral |
| G-CSF | tau | 5.71e–4 | -0.045 | -0.092 | -0.050 | -0.004 | -0.117 | -0.060 |
|  | BF_10_ | 0.168 | 0.190 | 0.286 | 0.196 | 0.167 | 0.399 | 0.210 |
|  | u 95% CI | 0.166 | 0.122 | 0.076 | 0.118 | 0.160 | 0.190 | 0.108 |
|  | l 95% CI | -0.168 | -0.210 | -0.255 | -0.216 | -0.170 | -0.112 | -0.224 |
| IL-1β | tau | 0.018 | 7.47e–4 | -0.122 | -0.005 | -0.005 | 0.041 | -0.129 |
|  | BF_10_ | 0.157 | 0.151 | 0.485 | 0.153 | 0.152 | 0.173 | 0.546 |
|  | u 95% CI | 0.168 | 0.150 | 0.032 | 0.146 | 0.144 | 0.190 | 0.028 |
|  | l 95% CI | -0.134 | -0.150 | -0.269 | -0.158 | -0.156 | -0.112 | -0.277 |
| IL-2 | tau | 0.089 | 0.043 | -0.059 | 0.070 | 0.056 | -0.073 | -0.243 |
|  | BF_10_ | 0.275 | 0.187 | 0.208 | 0.227 | 0.203 | 0.235 | 6.77 |
|  | u 95% CI | 0.251 | 0.204 | 0.108 | 0.231 | 0.218 | 0.094 | -0.066 |
|  | l 95% CI | -0.082 | -0.126 | -0.222 | -0.100 | -0.114 | -0.236 | -0.399 |
| IL-5 | tau | 0.200 | 0.067 | 0.106 | 0.167 | 0.018 | -0.010 | -0.144 |
|  | BF_10_ | 2.099 | 0.221 | 0.340 | 0.975 | 0.169 | 0.166 | 0.624 |
|  | u 95% CI | 0.355 | 0.229 | 0.265 | 0.323 | 0.180 | 0.154 | 0.028 |
|  | l 95% CI | 0.024 | -0.102 | -0.064 | -0.008 | -0.148 | -0.174 | -0.305 |
| IL-6 | tau | 0.112 | 0.008 | -0.053 | 0.053 | 0.001 | -0.067 | -0.136 |
|  | BF_10_ | 0.401 | 0.153 | 0.189 | 0.190 | 0.152 | 0.216 | 0.626 |
|  | u 95% CI | 0.261 | 0.158 | 0.100 | 0.202 | 0.152 | 0.086 | 0.022 |
|  | l 95% CI | -0.046 | -0.146 | -0.204 | -0.102 | -0.152 | -0.218 | -0.285 |
| IL-7 | tau | -0.034 | -0.061 | -0.073 | -0.034 | 0.107 | -0.034 | -0.012 |
|  | BF_10_ | 0.168 | 0.203 | 0.230 | 0.168 | 0.367 | 0.168 | 0.156 |
|  | u 95% CI | 0.120 | 0.094 | 0.082 | 0.120 | 0.255 | 0.118 | 0.142 |
|  | l 95% CI | -0.186 | -0.212 | -0.224 | -0.186 | -0.050 | -0.186 | -0.166 |
| IL-8 | tau | 0.025 | 0.033 | -0.028 | 0.023 | -0.011 | 0.038 | -0.052 |
|  | BF_10_ | 0.156 | 0.161 | 0.158 | 0.155 | 0.149 | 0.166 | 0.186 |
|  | u 95% CI | 0.170 | 0.178 | 0.118 | 0.168 | 0.136 | 0.182 | 0.096 |
|  | l 95% CI | -0.124 | -0.116 | -0.174 | -0.126 | -0.158 | -0.112 | -0.198 |
| IL-10 | tau | 0.130 | -0.074 | -0.035 | 0.028 | 0.182 | -0.084 | -0.139 |
|  | BF_10_ | 0.407 | 0.251 | 0.208 | 0.207 | 0.835 | 0.269 | 0.355 |
|  | u 95% CI | 0.319 | 0.126 | 0.162 | 0.224 | 0.365 | 0.116 | 0.068 |
|  | l 95% CI | -0.078 | -0.167 | -0.231 | -0.174 | -0.028 | -0.277 | -0.331 |
| IL-12 (p70) | tau | 0.186 | 0.075 | 0.070 | 0.156 | 0.114 | -0.018 | -0.113 |
|  | BF_10_ | 2.54 | 0.235 | 0.222 | 1.07 | 0.429 | 0.152 | 0.419 |
|  | u 95% CI | 0.327 | 0.218 | 0.214 | 0.297 | 0.254 | 0.128 | 0.038 |
|  | l 95% CI | 0.030 | -0.076 | -0.080 | 0.002 | -0.038 | -0.166 | -0.257 |
| IL-13 | tau | 0.012 | 0.050 | -0.008 | 0.029 | 0.022 | 0.027 | -0.068 |
|  | BF_10_ | 0.154 | 0.184 | 0.152 | 0.163 | 0.158 | 0.160 | 0.218 |
|  | u 95% CI | 0.162 | 0.198 | 0.142 | 0.178 | 0.172 | 0.176 | 0.086 |
|  | l 95% CI | -0.142 | -0.104 | -0.160 | -0.124 | -0.130 | -0.126 | -0.218 |
| IL-17A | tau | 0.043 | -0.047 | -0.008 | 0.015 | -0.031 | -0.053 | -0.174 |
|  | BF_10_ | 0.178 | 0.181 | 0.154 | 0.157 | 0.165 | 0.190 | 1.474 |
|  | u 95% CI | 0.194 | 0.106 | 0.144 | 0.168 | 0.122 | 0.100 | -0.014 |
|  | l 95% CI | -0.114 | -0.198 | -0.162 | -0.140 | -0.184 | -0.204 | -0.321 |
| MCP-1 (MCAF) | tau | -0.193 | -0.092 | -0.075 | -0.177 | -0.005 | 3.34e–4 | -0.116 |
|  | BF_10_ | 3.113 | 0.299 | 0.236 | 1.923 | 0.148 | 0.148 | 0.449 |
|  | u 95% CI | -0.040 | 0.056 | 0.074 | -0.024 | 0.140 | 0.146 | 0.034 |
|  | l 95% CI | -0.335 | -0.236 | -0.220 | -0.319 | -0.152 | -0.148 | -0.261 |
| MIP-1β | tau | 0.089 | 0.049 | 0.004 | 0.094 | -0.004 | -0.021 | -0.125 |
|  | BF_10_ | 0.283 | 0.180 | 0.148 | 0.306 | 0.148 | 0.153 | 0.527 |
|  | u 95% CI | 0.233 | 0.192 | 0.150 | 0.239 | 0.142 | 0.126 | 0.026 |
|  | l 95% CI | -0.062 | -0.100 | -0.144 | -0.058 | -0.152 | -0.168 | -0.269 |
| TNF-α | tau | 0.033 | -0.033 | -0.145 | -0.054 | -0.029 | -0.031 | -0.205 |
|  | BF_10_ | 0.162 | 0.162 | 0.840 | 0.189 | 0.158 | 0.160 | 4.596 |
|  | u 95% CI | 0.178 | 0.114 | 0.006 | 0.094 | 0.118 | 0.116 | -0.050 |
|  | l 95% CI | -0.1818 | -0.180 | -0.289 | -0.200 | -0.176 | -0.178 | -0.347 |

*Data was log-transformed prior to analysis*

References

Akdis, M., Aab, A., Altunbulakli, C., Azkur, K., Costa, R. A., Crameri, R., Duan, S., Eiwegger, T., Eljaszewicz, A., Ferstl, R., Frei, R., Garbani, M., Globinska, A., Hess, L., Huitema, C., Kubo, T., Komlosi, Z., Konieczna, P., Kovacs, N., … Akdis, C. A. (2016). Interleukins (from IL-1 to IL-38), interferons, transforming growth factor β, and TNF-α: Receptors, functions, and roles in diseases. *The Journal of Allergy and Clinical Immunology*, *138*(4), 984–1010. https://doi.org/10.1016/j.jaci.2016.06.033

Cameron, M. J., & Kelvin, D. J. (2013). Cytokines, Chemokines and Their Receptors. In *Madame Curie Bioscience Database [Internet]*. Landes Bioscience. https://www.ncbi.nlm.nih.gov/books/NBK6294/

Charles A Janeway, J., Travers, P., Walport, M., & Shlomchik, M. J. (2001). Induced innate responses to infection. In *Immunobiology: The Immune System in Health and Disease. 5th edition*. Garland Science. https://www.ncbi.nlm.nih.gov/sites/books/NBK27122/

Deshmane, S. L., Kremlev, S., Amini, S., & Sawaya, B. E. (2009a). Monocyte chemoattractant protein-1 (MCP-1): An overview. *Journal of Interferon & Cytokine Research: The Official Journal of the International Society for Interferon and Cytokine Research*, *29*(6), 313–326. https://doi.org/10.1089/jir.2008.0027

Deshmane, S. L., Kremlev, S., Amini, S., & Sawaya, B. E. (2009b). Monocyte Chemoattractant Protein-1 (MCP-1): An Overview. *Journal of Interferon & Cytokine Research*, *29*(6), 313–326. https://doi.org/10.1089/jir.2008.0027

Gaffen, S. L. (2008). An overview of IL-17 function and signaling. *Cytokine, Special Issue – Abstracts and Reviews: 7thJoint Conference of the International Cytokine Society and the International Society for Interferon and Cytokine Research*, *43*(3), 402–407. https://doi.org/10.1016/j.cyto.2008.07.017

Garbers, C., Heink, S., Korn, T., & Rose-John, S. (2018). Interleukin-6: Designing specific therapeutics for a complex cytokine. *Nature Reviews. Drug Discovery*, *17*(6), 395–412. https://doi.org/10.1038/nrd.2018.45

Greenfeder, S., Umland, S. P., Cuss, F. M., Chapman, R. W., & Egan, R. W. (2001). Th2 cytokines and asthma. The role of interleukin-5 in allergic eosinophilic disease. *Respiratory Research*, *2*(2), 71–79. https://doi.org/10.1186/rr41

Harris, A., & Patel, P. (2025). Interleukin. In *StatPearls [Internet]*. StatPearls Publishing. https://www.ncbi.nlm.nih.gov/sites/books/NBK499840/

Kurzrock, R. (2003). Granulocyte colony-stimulating factor. In *Holland-Frei Cancer Medicine. 6th edition*. BC Decker. https://www.ncbi.nlm.nih.gov/books/NBK12664/

Pelaia, C., Paoletti, G., Puggioni, F., Racca, F., Pelaia, G., Canonica, G. W., & Heffler, E. (2019). Interleukin-5 in the Pathophysiology of Severe Asthma. *Frontiers in Physiology*, *10*, 1514. https://doi.org/10.3389/fphys.2019.01514

Sabat, R., Grütz, G., Warszawska, K., Kirsch, S., Witte, E., Wolk, K., & Geginat, J. (2010). Biology of interleukin-10. *Cytokine & Growth Factor Reviews*, *21*(5), 331–344. https://doi.org/10.1016/j.cytogfr.2010.09.002

Tomiita, M., Campos-Alberto, E., Shima, M., Namiki, M., Sugimoto, K., Kojima, H., Watanabe, H., Sekine, K., Nishimuta, T., Kohno, Y., & Shimojo, N. (2015). Interleukin-10 and interleukin-5 balance in patients with active asthma, those in remission, and healthy controls. *Asia Pacific Allergy*, *5*(4), 210. https://doi.org/10.5415/apallergy.2015.5.4.210

Yang, X., Wang, S., Fan, Y., & Han, X. (2000). IL-10 deficiency prevents IL-5 overproduction and eosinophilic inflammation in a murine model of asthma-like reaction. *European Journal of Immunology*, *30*(2), 382–391. https://doi.org/10.1002/1521-4141(200002)30:2%3C382::AID-IMMU382%3E3.0.CO;2-L
